# Interactions between m6A modification and miRNAs in malignant tumors

**DOI:** 10.1038/s41419-021-03868-5

**Published:** 2021-06-09

**Authors:** Xiao Han, Jing Guo, Zhipeng Fan

**Affiliations:** 1 Beijing Key Laboratory of Tooth Regeneration and Function Reconstruction, Beijing Stomatological Hospital, School of Stomatology, Capital Medical University, Beijing, 100050 China; 2 School of Stomatology, Nanchang University, The Key Laboratory of Oral Biomedicine, Nanchang, Jiangxi Province China; 3 Research Unit of Tooth Development and Regeneration, Chinese Academy of Medical Sciences, Nanchang, Jiangxi Province China

**Keywords:** Biomarkers, Cancer

## Abstract

Recently, the regulatory role of epigenetic modifications in the occurrence and development of malignant tumors has attracted extensive attention. RNA m6A methylation is the most abundant RNA modification in eukaryotic cells and regulates RNA transcription, processing, splicing, degradation, and translation. As important biomarkers, miRNAs play a crucial role in the diagnosis and treatment of diseases as well as in the development of anti-tumor drugs. Recently, increasing evidence has shown that m6A modification plays a vital role in regulating miRNA biosynthesis. We, herein, have reviewed the enzyme system involved in m6A methylation and the crosstalk between m6A modification and miRNAs in cancer. In addition, we have discussed the potential clinical applications and possible development directions of this field in the future.

## Facts

- N6-methyladenosine regulates miRNA biosynthesis.
- miRNAs affect m6A levels by targeting m6A regulatory proteins.
- The interactions between m6A modification and miRNAs influence cancer progression.

## Open questions

- How does m6A inhibit the biological functions of miRNAs?
- Are there other m6A readers that recognize m6A modifications to perform different biological functions?
- Could the combination of m6A and miRNAs effectively treat cancer, especially drug-resistant cancer?

## Introduction

Recently, the regulatory role of epigenetic modifications in the occurrence and development of malignant tumors has attracted much attention. RNA epigenetics is an important way to regulate gene expression at the RNA level. The reversibility of epigenetics provides a scientific basis for early intervention and treatment of diseases. To date, more than 170 RNA modifications have been identified, and methylation modification is known to be the most common^1^. Studies have found that the RNA methylation modifications usually include N1-methyladenosine (m1A), 5-methylcytosine (m5C), 5-hydroxymethylcytosine (5hmC), N6-methyladenosine (m6A), and 7-methylguanine (m7G)^2^. Among them, m6A modification is the most abundant and important mRNA modification in mammals, and accounts for ~50% of the total methylated ribonucleotides^3^. Sequencing analysis showed that m6A modification was mainly concentrated in the common motif RRACH (R = G/A, H = A/C/U). In the RRACH motif, m6A modification is preferentially concentrated near the 3′-UTR, followed by the CDs and 5′-UTR regions^4,5^. In the 3′-UTRs enriched with m6A, about 67% of UTRs also contain ncRNAs, such as the binding sites of miRNAs, suggesting that m6A and ncRNAs may jointly regulate target mRNAs through cooperation or competition^4^. m6A modification is involved in the regulation of RNA metabolism, including translation, splicing, and degradation, and plays a crucial regulatory role in the development and disease onset and progression^6^.

m6A modification exists not only in mRNA but also in non-coding RNAs, such as miRNAs. miRNAs are noncoding single-stranded small RNA molecules with a length of ~21–23 nucleotides. They are widely found in eukaryotes and play an important role in post-transcriptional regulation of gene expression through translation inhibition and mRNA cleavage^7,8^. As an important biomarker, miRNA has a very important guiding significance in the diagnosis and treatment of diseases and development of antitumor drugs. m6A has been shown to be abnormally expressed in many tumors and plays a vital role in the regulation of a series of malignant biological behaviors such as cell proliferation, invasion, and metastasis. With the wide application of high-throughput sequencing technology and bioinformatics analysis in scientific research, increasing number of m6A-modified miRNAs have been discovered. Subsequently, researchers have also analyzed the interaction between m6A RNA methylation and miRNAs in the context of cancer. We, in this article, have reviewed the research progress regarding the interactions between m6A modification and miRNA in tumors; our aim was to provide a theoretical basis for a deeper understanding of the occurrence and development of malignant tumors as well as that regarding the search for tumor prediction biomarkers and therapeutic targets.

### m6A enzyme system

**Fig. 1:**
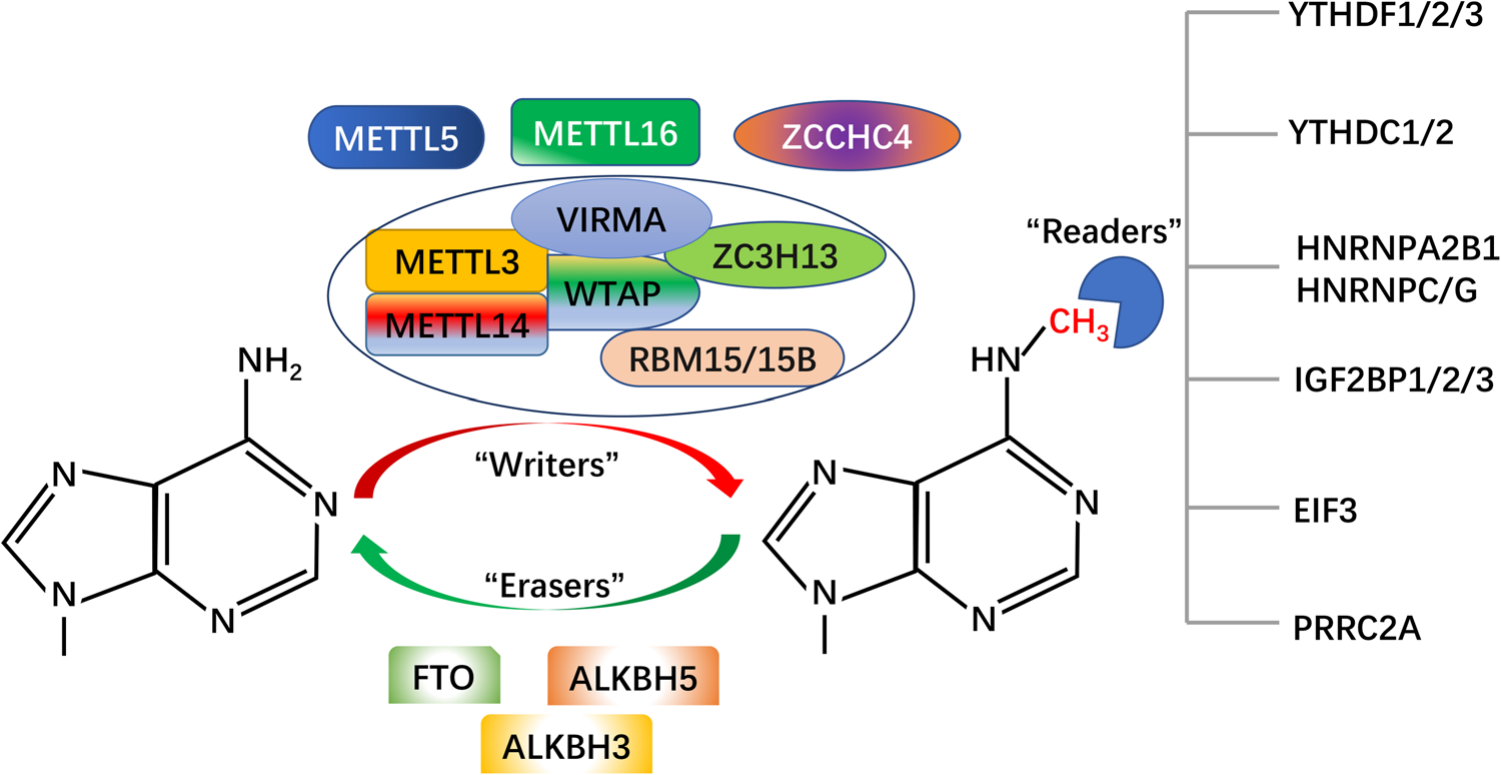
Dynamic reversible process and molecular composition of m6A. m6A modifications are catalyzed by m6A writers, erasers, and readers. The sixth N position of adenylate (A) can be methylated by m6A writers to form N6-methyladenosine (m6A). Writers include METTL3, METTL14, WTAP, RBM15/15B, VIRMA, and ZC3H13. METTL5, METTL16, and ZCCHC4 are independent m6A methyltransferases. Erasers are proteins with demethylase activity, and include FTO, ALKBH5, and ALKBH3. Readers are proteins that perform a biological function by recognizing m6A modifications, and include YTH family, HNRNP family, EIF3, IGF2BPs, and PRRC2A.

m6A refers to the methylation modification at the sixth N position of adenosine, and has been proven to be reversible. There are three main types of enzymes involved in m6A modification: methylated transferase, demethylated transferase, and methylated recognition protein (Fig. 1).

### m6A methylated transferase

m6A methylation is catalyzed by the methyltransferase complex (MTC) known as “m6A writers”. MTC is composed of m6A-METTL complex (MAC) and m6A-METTL associated complex (MACOM)^9^. MAC consists of methyl transferase 3 (METTL3) and methyl transferase 14 (METTL14), which can form stable heterodimers. METTL3 is the first discovered methyltransferase with a catalytic subunit, and serves as the main catalytic core^10,11^. METTL14 can recognize substrates and promote binding to RNA as well as increase the activity of METTL3 methyltransferase^12,13^. MACOM consists of Wilms tumor-1 associated protein (WTAP), CCCH type 13 zinc finger protein (ZC3H13), m6A methyltransferase-associated virus (VIRMA), and RNA-binding protein 15/15b (RBM15/15b). WTAP itself does not have methyltransferase activity, but it can directly bind to METTL3 to ensure the proper localization of the METTL3–METTL14 heterodimer, thus improving the activity of methyltransferase^14^. ZC3H13 can promote nuclear localization of the complexes^15^. VIRMA recruits core catalytic components to mediate preferred methylation in the 3′-UTR^16^. RBM15/15b can bind complexes and recruit them to specific sites on RNA to promote methylation^17^. In addition to the above methyltransferase in the form of a complex, independent methyl transferases METTL16, METTL5, and zinc finger CCHC type containing 4 (ZCCHC4) have also been identified. METTL16 was observed to catalyze m6A methylation of U6-like hairpins in U6 snRNA and methionine adenosine transferase (MAT2A) as well as to regulate the variable splicing of MAT2A, thereby maintaining SAM’s homeostasis^18^. ZCCHC4 mediates m6A methylation of 28S rRNA, which plays an important role in the onset and development of tumors^19^. A recent study showed that METTL5 was able to mediate the methylation of 18S rRNA m6A and maintain the stability of cell metabolism by forming a heterodimer with TRMT112^20^. In summary, we, from the literature, have identified that m6A methyltransferase exists mainly in two forms. One is in the form of a methyltransferase complex, in which different methyltransferases perform their respective functions and eventually lead to m6A methylation modification of RNA. The other is independent of the presence of methyltransferase, independent of the form of the complex, but independent of methylation. Further research on “m6A writers” may provide novel biomarkers for tumor diagnosis as well as new directions for the discovery of tumor therapeutic targets.

### m6A demethylated transferase

m6A methylation is dynamically reversible and can be reversed by demethylases called “m6A Erasers”. Erasers mainly include FTO and ALKBH5, which belong to the ALKB family, with Fe (II) and alpha-ketone glutaric acid-dependent way of catalyzing m6A demethylation^9,21^. FTO is the first to be found to methylation enzyme; it can catalyze m6A oxidation of N6-hydroxymethyladenosine (hm6A) and N6-formyladenosine (f6A)^22^. Studies have found that FTO can also demethylate m6Am in mRNAs and snRNAs, and m1A in tRNAs^23–25^. ALKBH5 is the second discovered demethylase, and can directly reverse m6A to adenosine^26^. Recently, ALKBH3, as a demethylase, was found to be capable of removing the m6A modification of tRNAs^27^. The discovery of m6A dimethyl transferase is of great significance to the researchers analyzing m6A and can provide great help for reverse treatment. However, at present, our understanding of m6A dimethyl transferase is still in the exploratory stage and needs further analysis.

### m6A regulatory proteins

m6A modified RNAs require a class of m6A binding proteins that can specifically recognize and mediate their specific biological functions; these proteins known as “m6A readers”^9^. Readers are mainly divided into proteins containing the YTH domain and proteins without this domain. The YTH domain can selectively bind to the m6A site of RNA. The proteins containing this YTH domain include YTHDF1, YTHDF2, YTHDF3, YTHDC1, and YTHDC2^28^. YTHDF1-3 specifically recognize the mRNA containing m6A in the cytoplasm and promote mRNA translation and degradation^29–31^. YTHDC1-2 mainly recognize m6A-binding mRNA in the nucleus and promote mRNA degradation^32,33^. Heterogeneous nuclear ribonucleoproteins A2/B1 (HNRNPA2B1) and C (HNRNPC) of the nuclear heterogeneous ribonucleoprotein family are two rich nuclear RNA-binding proteins involved in the processing of precursor mRNA. HNRNPA2B1 binds to m6A modification sites on RNA to regulate RNA splicing and miRNA maturation^34^. HNRNPC and HNRNPG can regulate the alternative splicing of transcripts containing m6A modifications by recognizing and binding to m6A dependent structural switches^35,36^. Eukaryotic initiation factor 3 can directly bind to the 5′-UTR of m6A-modified mRNA, initiating mRNA translation^37^. The insulin-like growth factor-2 binding protein family (IGF2BPs, including IGF2BP1/2/3) promotes mRNA translation that is more stable in an m6A-dependent manner^38^. A new m6A reader, PRRC2A, was recently found to be dependent on m6A to stabilize mRNA expression^39^. Overall, these results indicate that numerous m6A readers have been found thus far, which shows that potentially numerous m6A readers with wide targeting abilities exist, thus offering a broad research space. Since m6A modification relies on “Readers” to perform its biological function; the same m6A modification may have opposite biological effects when combined with different “Readers”. Therefore, promoting or blocking the binding of m6A RNA to “Readers” may provide a new approach for cancer therapy in the future.

### Mutual regulation between m6A and miRNAs

#### m6A regulates the biosynthesis of miRNAs

**Fig. 2:**
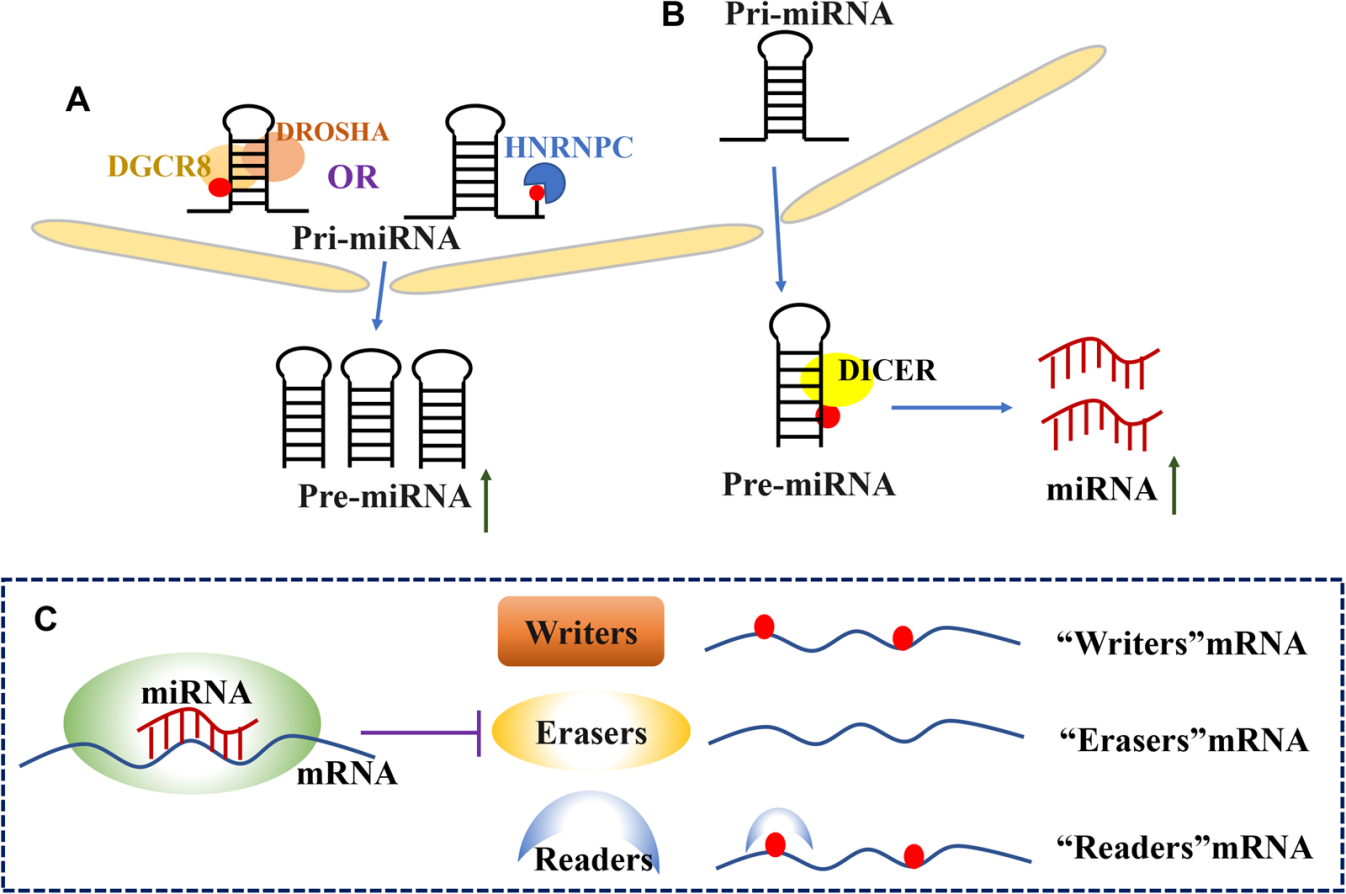
Mutual regulation between m6A and miRNAs. **A** In the nucleus, the intranuclear enzyme Drosha and DGCR8 cleave primary microRNA (pri-miRNA) to precursor microRNA (pre-miRNA). m6A modifications helps to recruit DGCR8 to target pri- miRNA and promote the formation of pre-miRNA. Meanwhile, HNRNPC can directly recognize the m6A sites. **B** In the cytoplasm, Dicer cleaves pre-miRNAs transported to the cytoplasm into mature miRNAs. **C** miRNAs regulate the expression of m6A regulatory proteins. miRNAs regulate the expression of mRNAs encoding for writers, erasers, and readers.

The classical miRNA synthesis route is as follows: first, miRNAs in the cell nucleus are transcribed into pri-miRNAs under the action of RNA polymerase II, and pri-miRNAs form pre-miRNAs under the action of a micro-processing complex composed of endonuclease Drosha and a cofactor, double-stranded RNA binding protein DGCR8. Pre-miRNAs are transported to the cytoplasm and then spliced by the endoribonuclease Dicer to form double-stranded microRNAs^7,8^. It has been found that m6A modification can regulate miRNA biosynthesis by participating in the processing of pri-miRNAs or splicing of pre-miRNAs (Fig. 2).

Alarcon et al. first discovered the regulation of methylase METTL3 in miRNA biosynthesis in 2015. Studies have shown that METTL3 induces m6A modification in pri-miRNAs and enhances DGCR8 recognition and binding to its substrate, thus promoting the processing of pri-miRNAs and miRNA maturation^40^. Recently, in addition to its interaction with DGCR8, METTL3 has been shown to promote the synthesis of mature miRNAs by increasing the splicing of pre-miRNAs by Dicer^41^. METTL14 can promote the processing of pri-miR-375 by regulating the m6A modification through interaction with DGCR8^42^. Subsequently, Berulava et al. found that the levels of pri-miRNAs were not altered by knocking down the methyltransferase FTO, however, homeostatic levels of some potential methylated miRNAs were reduced^43^. Nonetheless, the mechanism by which FTO negatively regulates the stability of miRNAs is still unclear and needs to be further investigated. The interactions between the demethylase ALKBH5 and dead box helicase family member DDX3 regulate the m6A level in mRNAs. DDX3 interacts with AGO2 to regulate the demethylation of miRNAs; however, whether there is a direct interaction between ALKBH5 and AGO2 needs further verification^44^. In addition, the NF-κB activator protein NKAP, which is recognized by m6A, promotes the processing of pri-miR-25 by interacting with DGCR8 to recognize and bind to the m6A site on pri-miR-25^45^. HNRNPC can directly recognize and bind to the pri-miR-21 site modified by m6A to promote the expression of miR-21^46^. HNRNPA2B1 is of particular interest as it has been shown to promote the processing of some of the pri-miRNAs by recruiting DGCR8. In addition, HNRNPA2B1 has been found to inhibit the shearing of some of the pri-miRNAs; however, the specific mechanism underlying this inhibition remains unclear^34,47^.

### miRNAs regulate m6A modifications

In the classical miRNA synthesis pathway, mature miRNAs bind to the 3′-UTR of their target mRNA through complementary base pairing, which leads to the degradation of target mRNA or inhibition of protein translation. m6A modifications can also be used as target genes of miRNAs (Fig. 2).

A study found that miR-33a can inhibit the proliferation and migration of cancer cells by directly binding to the 3′-UTR of *METTL3*, thus reducing the expression of METTL3^48^. Subsequently, He et al. found that miR-4429 can target and downregulate *METTL3* to inhibit the stabilization of *SEC62* induced by m6A. The results showed that *METTL3* can be used as a target gene by miRNAs and that it can regulate the mRNA stability of downstream genes through m6A modification^49^. Moreover, a study found that in hepatocellular carcinoma (HCC) cells, miR-145 regulated the level of m6A by targeting the 3′-UTR of *YTHDF2* mRNA^50^. Recent studies have found that YTHDF1 can act as a target of miR-346 to regulate the progression of glioma^51^.

In conclusion, on one hand, m6A modifications can promote the processing of pri-miRNAs through the interaction with RNA-binding protein DGCR8 or promote the maturation of miRNAs by increasing the splicing of pre-miRNAs by Dicer and positively regulating the synthesis and expression of miRNAs. On the other hand, m6A also plays a negative regulatory role in miRNA synthesis. For example, HNRNPA2B1 can inhibit the over-splicing of some of the pri-miRNAs; nonetheless, the specific inhibitory mechanism remains to be elucidated. In addition, miRNAs can directly target m6A-related proteins, resulting in the translation inhibition of m6A-related protein-encoding genes, thus playing a negative regulatory role.

### Interaction between m6A and miRNAs in cancer

**Fig. 3:**
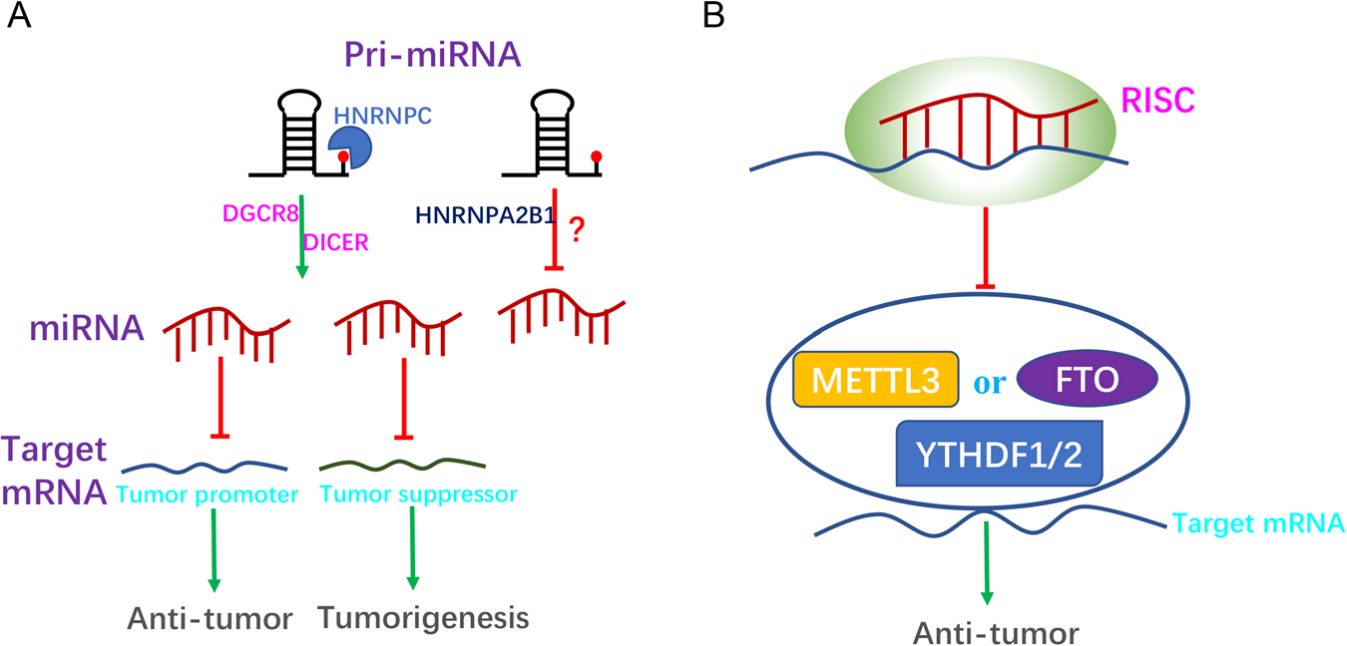
Interaction between m6A and miRNAs in cancer. **A** m6A modifications play different roles in multiple cancers by influencing the maturation of miRNAs. m6A modification promotes miRNA maturation by recruiting DGCR8 to promote pri-miRNA processing or increasing pre-miRNA splicing by Dicer in different cancers. Furthermore, m6A modification can promote or inhibit the occurrence and development of tumors by affecting the maturation of miRNAs. HNRNPA2B1 has also been found to inhibit the shearing of some of the pri-miRNAs; however, the specific mechanism underlying this inhibition remains unclear in breast cancer. **B** miRNAs affect cancer progression by regulating m6A modification of mRNAs (*METTL3, FTO, YTHDF1, and YTHDF2*).

In recent years, it has been found that m6A exists in miRNAs targeting oncogenes or tumor suppressors, and is involved in the onset and development of cancer by affecting the biogenesis or stability of miRNAs (Table 1 and Fig. 3).

**Table 1:** M6A regulate miRNAs in different cancers.

m6A Regulator	Cancer	miRNAs	Biological function	Ref.
METTL3	bladder	miR-221/222	Promote cells proliferation in bladder	^52^
METTL3	colorectal	miR-1246	Promote cells migration and invasion in CRC	^53^
METTL3	pancreatic	miR-25-3p	Promote cells proliferation migration and invasion in pancreatic cancer	^45^
METTL3	NSCLC	miR-143-3p	Promote cells invasion in NSCLC	^41^
Mettl14	breast	miR-146a-5p	Promote cells migration and invasion in breast cancer	^55^
Mettl14	hepatoma	miR-126	Suppress cells migration in HCC	^54^
Mettl14	colorectal	miR-375	Suppress cells proliferation invasion and migration in CRC	^42^
ALKBH5	ovarian	miR-7	Promote cells proliferation, invasion, and autophagy in ovarian cancer	^57^
NKAP	pancreatic	miR-25-3p	Promote cells proliferation migration and invasion in pancreatic cancer	^45^
HNRNPA2B1	NSCLC	miR-106b-5p	Promote cells proliferation in NSCLC	^58^
HNRNPC	glioblastoma	miR-21	Promote cells migration and invasion in glioblastoma	^46^

Previous studies have shown that METTL3 promotes the processing of pri-miRNAs by recruiting DGCR8 to identify substrates^40^. As of 2019, this mechanism has been confirmed in various human cancers. As reported by Han et al., METTL3 expression was found to be upregulated in bladder cancer tissues and cell lines. Furthermore, METTL3 promoted the maturation of miR-221/222 in bladder cancer cells by recruiting DGCR8. The expression of miR-221/222 target gene *PTEN* was downregulated, which promoted the proliferation of bladder cancer cells. The study showed that METTL3 may act as a new prognostic marker for the treatment of bladder cancer^52^. Subsequently, Peng et al. found a similar mechanism involving METTL3 in colorectal cancer (CRC). METTL3 promoted the biogenesis of miR-1246 by interacting with DGCR8, which in turn promoted the migration and invasion of CRC cells. miR-1246 was found to reverse the inhibition of MAPK signaling pathways by downregulating the tumor suppressor gene *SPRED2*, which enhanced the metastatic ability of CRC^53^. Moreover, another study found that cigarette condensate can induce DNA hypomethylation in *METTL3* promoter, which promotes the expression of METTL3, resulting in m6A modification of pri-miR-25, followed by enhancing the processing of pri-miR-25 in NKAP-dependent manner. Mature miR-25-3p promoted the proliferation, migration, and invasion of pancreatic cancer cells by inhibiting PHLPP2 and activating the oncogenic AKT-p70S6K signaling pathway^45^. METTL3 has also been shown to promote miRNA biogenesis by increasing the splicing of pre-miRNAs by Dicer. Recently, a study found that miR-143-3p was highly expressed in lung cancer patients with brain metastasis, which depended on METTL3 promoting Dicer splicing pre-miR-143 into mature miR-143 in lung cancer cells, followed by downregulation of VASH1-dependent degradation of VEGF, which promoted angiogenesis in lung cancer patients with brain metastasis. This was the first study to reveal the role of m6A in pre-RNA processing, thus providing a new therapeutic target for patients with non-small cell lung cancer (NSCLC) brain metastases^41^. In summary, the results show that METTL3 promotes the occurrence and development of tumors by enhancing the synthesis of tumor-promoting factor miRNAs in bladder, colorectal, pancreatic, and lung cancers. METTL14 plays a similar role in cancer as that of METTL3; it promotes DGCR8 to recognize and bind m6A-modified pri-miRNAs, which further mediates miRNA maturation. Studies have shown that METTL14 promotes pri-miR-126 maturation through the recruitment of DGCR8-dependent m6A in HCC. Deletion of *METTL14* in HCC cells reduces m6A levels and the expression of miR-126, leading to the migration and invasion of cancer cells^54^. Another study found that METTL14 inhibited the progression of CRC by promoting the maturation of miR-375 in an m6A-dependent manner. It not only inhibited the proliferation of cancer cells through miR-375/YAP1, but also inhibited their migration and invasion via the miR-375/SP1 pathway^42^. Yi et al. observed abnormal expression of METTL14 in breast cancer tissues and cells, and further found that overexpression of METTL14 promoted the migratory and invasive abilities of breast cancer cells by promoting the expression of miR-146a-5p^55^. In summary, the results indicate that METTL14 can inhibit or promote the occurrence of tumors by regulating the synthesis of miRNAs. In HCC and CRC, METTL14 inhibits the occurrence of tumors by promoting the synthesis of tumor-suppressor miRNAs, whereas in breast cancer, METTL14 promotes the occurrence and development of tumors by enhancing the synthesis of tumor-promoting miRNAs.

The role of the demethylase enzyme FTO in regulating miRNAs in cancer has not been reported till date. However, Shen et al. found that FTO plays an important role as a downstream target gene of miR-1266 in colorectal cancer and that miR-1266 directly targets the 3′-UTR of *FTO* to inhibit the proliferation of colorectal cancer cells^56^. Another demethylase, ALKBH5, promoted the maturation of miR-7 in a hub-dependent manner and upregulated the mRNA levels of *Bcl2* in m6A-dependent manner, thus promoting the proliferation, migration, and autophagy of ovarian cancer cells^57^.

Furthermore, the m6A reader participates in miRNA biogenesis. HNRNPA2B1 has been shown to promote miRNA synthesis by recruiting DGCR8 to recognize the m6A marker site of pri-miRNAs^34^. Consistent with this finding, the overexpressed oncogene *LINC01234* in NSCLC promotes maturation from pri-miR-106b to miR-106b-5p by interacting with hnRNPA2B1 to recruit DGCR8, resulting in a decrease in cryptochrome 2 as well as promotion of c-MYC expression. Interestingly, activated c-MYC binds to the promoter of *LINC01234* and increases its transcription, thus forming a positive feedback loop that leads to cell proliferation and tumor growth^58^. However, the role of HNRNPA2B1 in the regulation of miRNAs appears to be complex in breast cancer. Genome-wide miRNA sequencing was performed post the upregulation of HNRNPA2B1 in endocrine-resistant breast cancer cells. Sequencing results showed that miR-1266-5p, miR-1268a, and miR-671-3p were upregulated in the HNRNPA2B1 group, while miR-29a-3p, miR-29b-3p, and miR-222 were downregulated in this group. However, the biological processes by which HNRNPA2B1 inhibits miRNAs are not yet clear^47^. In summary, these results suggest that HNRNPA2B1 may act as an oncogenic factor in NSCLC; however, its specific role in breast cancer remains unclear and further research is needed in this direction. In glioblastoma, HNRNPC can directly recognize and bind to pri-miR-21 sites that have been modified with m6A and promote the expression of miR-21. Silencing HNRNPC in glioblastoma cells reduced the level of miR-21, thereby increasing the expression of PDCD4, inhibiting the activation of Akt and p70S6K, and inhibiting cell migration and invasion^46^.

m6A modifications have also been found to play an important role in cancer progression by acting as targets for miRNAs (Table 2 and Fig. 3). In NSCLC, the expression level of METTL3 was higher in lung cancer than in normal tissues. Moreover, miR-33a inhibited the migration and proliferation of lung cancer cells by directly targeting the 3′-UTR of *METTL3* and decreasing its mRNA levels^48^. In addition, in NSCLC, miR-600 induced the mitochondrial apoptosis signaling pathway by downregulating METTL3 expression to promote apoptosis and inhibit proliferation and invasion of lung cancer cells^59^. In breast cancer, let-7g, as a tumor suppressor, was found to downregulate the expression of METTL3 by targeting the 3′-UTR of *METTL3* mRNA, while hepatitis B virus X-interacting protein (HBXIP) could increase the expression of METTL3 by inhibiting the function of let-7g. Interestingly, METTL3 upregulated HBXIP in an m6A dependent manner, forming a positive feedback loop of HBXIP/let-7g/METTL3/HBXIP, and in turn, accelerating the proliferation and invasion of breast cancer cells^60^. In gastric cancer, miR-4429 was observed to target and reduce the mRNA expression of *METTL3*, inhibiting the stability of SEC62 induced by m6A, thereby inhibiting the proliferation and inducing apoptosis of gastric cancer cells^49^. In hepatoblastoma (HB) cells, knockout of METTL3 notably inhibited the proliferation, migration, and invasion of cells. Furthermore, miR-186 suppressed the progression of HB by directly targeting the 3′-UTR of METTL3 and activating the Wnt/β-catenin signaling pathway^61^. Collectively, these findings indicate that METTL3 can play a tumor-promoting role in NSCLC, gastric cancer, hepatoblastoma, and breast cancer.

**Table 2:** MiRNAs regulate m6A key proteins in different cancers.

miRNAs	Cancer	mRNAs	Biological function	Ref.
miR-33a	NSCLC	METTL3	Suppress cells proliferation in NSCLC	^48^
Let-7g	breast	METTL3	Suppress cells proliferation in breast cancer	^60^
miR-600	NSCLC	METTL3	Suppress cells proliferation migration and promote apoptosis in NSCLC	^59^
miR-4429	gastric	METTL3	Suppress cells proliferation induce apoptosis in gastric cancer	^49^
miR-186	hepatoma	METTL3	Suppress cells proliferation invasion and migration in hepatoma	^61^
miR-1266	colorectal	FTO	Suppress cells proliferation in colorectal cancer	^56^
miR-346	glioblastoma	YTHDF1	Suppress cells proliferation and tumor growth in glioblastoma	^51^
miR-145	hepatoma	YTHDF2	Suppress cells proliferation in hepatoma	^50^
miR-493-3p	prostate	YTHDF2	Suppress cells proliferation and migration in prostate cancer	^62^
miR-744-5p	ovarian	HNRNPC	Promote cells apoptosis in ovarian cancer	^63^

**Fig. 4:**
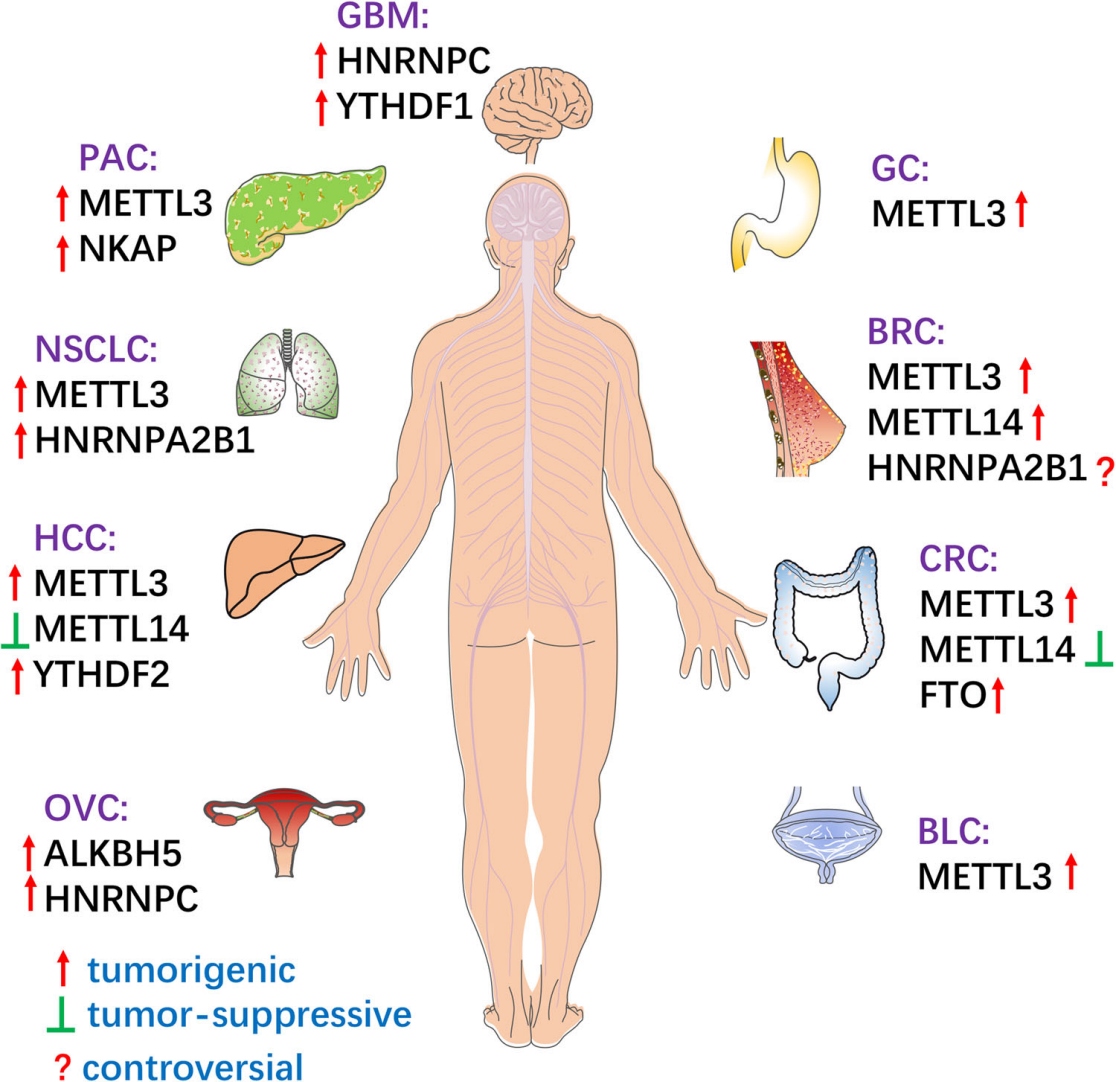
m6A modifications in different human cancers. m6A modifications participate in tumorigenesis and metastasis in different cancers. BLC, bladder cancer; CRC, colorectal cancer; BRC, breast cancer; GBM, glioblastoma; GC, gastric cancer; PAC, pancreatic cancer; NSCLC, non-small cell lung cancer; HCC, hepatocellular carcinoma; OVC, ovarian cancer.

miRNAs can participate in cancer progression by targeting the mRNA of m6A regulatory proteins. Studies have shown that miR-493-3p can directly target YTHDF2 and downregulate its expression, and reduce the level of m6A to inhibit the proliferation and invasion of prostate cancer cells^62^. In addition, YTHDF2 was found to be a downstream target of miR-145, which is involved in the regulation of m6A levels as well as promotion of the proliferation of HCC cells^50^. In glioblastoma, miR-346 targeting the 3′-UTR of YTHDF1 reduced the mRNA level of *YTHDF1*, inhibiting cell proliferation and tumor growth^51^. In ovarian cancer cells, HNRNPC was found to be a target of miR-744-5p, which is known to induce apoptosis. Furthermore, silencing HNRNPC could inhibit the mRNA levels of miR-21 and decrease the phosphorylation of the Akt ser473 site, thus promoting the apoptosis of ovarian cancer cells^63^. As mentioned above, in a variety of cancers, m6A modification is regulated by different m6A regulatory proteins by facilitating miRNA biosynthesis, and miRNAs, in turn, regulate the biological functions of m6A regulatory proteins (Fig. 4).

## Conclusion

In conclusion, m6A methylation modification and miRNAs play an important role in the regulation of tumor development. On one hand, m6A modification controls tumorigenesis by affecting miRNA maturation, while on the other hand, m6A related proteins can be used as downstream targets of miRNAs to participate in the development of tumors. Therefore, it is important to elucidate the role of miRNAs and m6A modifications in the regulation of their interrelationship in tumor therapy for all cancer types. However, current studies mostly focus on the abnormal expression of m6A modifications. Furthermore, the molecular mechanism and downstream signaling pathways involved in the differential transcriptome regulation of m6A modification-related proteins between tumor and normal cells require further analysis. In addition, the current studies on the regulatory mechanism of m6A are mostly limited to in vitro analyses, while only few relevant in vivo studies have been carried out. At the same time, due to the complexity and diversity of m6A modifications and their wide presence in many aspects of life activities, it is still a huge challenge to find translational applications of results obtained from research pertaining to m6A modifications. More research is required in the future to further understand the underlying mechanisms as well as to elucidate the regulatory relationship between m6A modification and miRNAs in tumors. With the newer insights, analysis of m6A methylation modification may lead to novel future directions for the effective management of a variety of diseases. Alone or in combination with miRNAs, it may have a strong therapeutic potential for the treatment of various types of cancer, especially for those with drug resistance.
